# A novel form of transcutaneous electrical nerve stimulation for the reduction of dysesthesias caused by spinal nerve dysfunction: A case series

**DOI:** 10.3389/fnhum.2022.937319

**Published:** 2022-08-24

**Authors:** Yuki Nishi, Koki Ikuno, Yuji Minamikawa, Yuki Igawa, Michihiro Osumi, Shu Morioka

**Affiliations:** ^1^Institute of Biomedical Sciences (Health Sciences), Nagasaki University, Nagasaki, Japan; ^2^Neurorehabilitation Research Center, Kio University, Nara, Japan; ^3^Department of Rehabilitation Medicine, Nishiyamato Rehabilitation Hospital, Nara, Japan; ^4^Graduate School of Health Science, Kio University, Nara, Japan

**Keywords:** dysesthesia, transcutaneous electrical nerve stimulation, spinal cord dysfunction, sensation, allodynia

## Abstract

**Background:**

Current therapeutic interventions for dysesthesias caused by spinal cord dysfunctions are ineffective. We propose a novel intervention using transcutaneous electrical nerve stimulation (TENS) for dysesthesias, and we present an in-depth case series.

**Patients and methods:**

Conventional high-frequency TENS and the novel dysesthesia-matched TENS (DM-TENS) were applied to 16 hands of nine patients with spinal cord dysfunction. The dysesthesia-matched TENS’ stimulus intensity and frequency matched the intensity and somatosensory profile of the patients’ dysesthesias. The Short-Form McGill Pain Questionnaire version-2 (SF-MPQ2) and quantitative sensory testing (QST) were applied during electrical stimulation/no stimulation. We determined intraclass correlation coefficients (ICCs) to evaluate the reliability of the setting and the effects on the dysesthesias and the change in subjective dysesthesia between each patient’s baseline without TENS and DM-TENS.

**Results:**

We were able to apply electrical stimulation matching the patients’ subjective dysesthesia for 14 hands (eight patients). TENS could not be applied for the remaining patient due to severe sensory deficits. Compared to the patients’ baseline and high-frequency TENS, the DM-TENS provided significant decreases in tingling/pins-and-needles and numbness on the SF-MPQ2, and it significantly improved the dynamic and static mechanical detection on QST. Regarding the reliability of the dysesthesia-matched TENS settings, the ICCs (1,5) were intensity, 0.95; frequency, 1.00; and effect on dysesthesia, 0.98.

**Conclusion:**

DM-TENS improved the dysesthesias and mechanical hypoesthesia caused by spinal cord dysfunction. The effectiveness of DM-TENS particularly for tingling and numbness was clearly higher and was reliable within the patients. These results may suggest an effective treatment of dysesthesias in patients with spinal cord dysfunction.

**Clinical trial registration:**

[https://rctportal.niph.go.jp/s/detail/um?trial_id=UMIN000045332], identifier [UMIN000045332].

## Introduction

Spinal cord dysfunction (e.g., spinal cord injury, cervical spondylotic myelopathy, and transverse myelitis) frequently cause dysesthesia in one or both upper limbs, resulting in reduced physical-activity functions and quality of life (Kokubun et al., 1996; Finnerup et al., 2001; Liu et al., 2015). In these conditions, dysesthesia is often complicated by neuropathic pain and somatosensory deficit, which interact with each other (Berić et al., 1988). Dysesthesia is a generic term for one or more unpleasant abnormal sensations in the extremities or other parts of body, whether spontaneous or evoked (Merskey and Bogduk, 1994). Spontaneous dysesthesias are described with terms such as tingling, pricking, numbness, “pins and needles,” tickling, and/or a “crawling” sensation. These abnormal sensations are rhythmic sensations perceived at regular intervals, and the rhythms depend on individual differences. Allodynia is an evoked form of dysesthesia in which normally painless stimuli are perceived as painful regardless of the specificity of the sensory modality, whereas hyperalgesia increases the response to a stimulus that is normally painful (Merskey and Bogduk, 1994).

These diverse abnormal sensations reflect the pathophysiological mechanisms in injured and surviving afferent nerve fibers, including ectopic impulse generation, conduction block, and peripheral and central sensitization (Campbell and Meyer, 2006). For example, although the ascending pathways and the brain areas that are involved in the dysesthesias are similar to pain mechanisms, the pricking or tingling sensations involve Aβ nerve fibers and have shown unique activation characteristics in several brain regions (Tihanyi et al., 2018; Wang et al., 2021). Several different neural mechanisms for allodynia have been identified that depend on the type of somatosensory modality (von Hehn et al., 2012). Analyses of individual somatosensory profiles could thus reveal important clues regarding the abnormal afferent processing of underlying dysesthesia (Baron et al., 2010).

Systematic reviews revealed that pharmacological treatments have been effective as therapeutic interventions for dysesthesias, but the degree of improvement was relatively low, and the risk of adverse events was high (Teasell et al., 2010; Snedecor et al., 2013). As a non-pharmacologic treatment, transcutaneous electrical nerve stimulation (TENS) is a safe and inexpensive therapy for neuropathic pain (Kopsky et al., 2014; Sluka, 2016). The effects of TENS on allodynia and hyperalgesia have been described (Tong et al., 2007; DeSantana et al., 2008). TENS affects the neuronal hyperexcitability underlying allodynia and hyperalgesia by promoting the descending inhibition of primarily dorsal horn wide-dynamic-range neurons in the central nervous system (CNS) (Witting et al., 2003; Meyer-Frießem et al., 2019). However, the effect of TENS on allodynia and hyperalgesia have been limited, and the effects on tingling and numbness are unclear. It has been noted that one of the factors associated with limited treatment effects of TENS could be related to the use of inappropriate parameters, the choice of which could result in different neurophysiological responses (Sluka and Walsh, 2003). In addition, dysesthesias have not been quantitatively assessed, and in-depth case studies have not been reported.

In this study, we propose a novel intervention method using TENS for the dysesthesias experienced in spinal cord dysfunction diseases, and we present an in-depth case series study. We refer to reports that electrical stimulation can provide a relatively stable variety of near-natural sensory information when the stimulus intensity and the frequency of TENS are set to match the intensity and the somatosensory profile of the dysesthesias (Tan et al., 2014; Slopsema et al., 2018). We speculate that this dysesthesia-matched TENS (DM-TENS) may improve dysesthesia by blocking dysesthesia-specific nerve fibers in the CNS. We investigated the short-term effects of DM-TENS in a variety of patients with dysesthesia due to spinal cord dysfunction in a comparison of pre-treatment and treatment data with conventional high-frequency TENS at settings described in previous studies. We hypothesized that (i) there is a case-specific intensity and frequency of electrical stimulation that matches an individual’s dysesthesia profile, and (ii) the new DM-TENS would be more effective against dysesthesia compared to TENS at the settings used in previous studies.

## Patients and methods

### Patients

Nine patients with spinal cord dysfunction who had dysesthesia were recruited at Nishiyamato Rehabilitation Hospital during the period from September 2021 to January 2022. The selection criteria were as follows: (1) diagnosis of spinal cord dysfunction such as spinal cord injury, cervical spondylotic myelopathy, myelitis, or atlanto-axial subluxation; (2) dysesthesia duration > 3 months; (3) a score ≥ 3 points on an 11-point numeric rating scale (NRS, 0–10) for dysesthesia intensity; and (4) American Spinal Injury Association (ASIA) Impairment Scale category C or D. The exclusion criteria were: (1) history of peripheral arterial disease, diabetes, skin disorder, peripheral neuropathy, metabolic disorder, hyperventilation syndrome, amyotrophic lateral sclerosis, Parkinson’s disease, autonomic neuropathy, or restless legs syndrome that may cause dysesthesia; and (2) a score < 24 on the Mini-Mental State Examination. The study protocol conformed to the Declaration of Helsinki. Each patient provided written informed consent before participating. The study was approved by the Institutional Ethics Board of Nishiyamato Rehabilitation Hospital (approval no. 0031) and was registered in the UMIN Clinical Trials Registry (#UMIN000045332).

### Experimental procedures

For the assessment of the patients’ spinal cord injury symptoms, each patient was examined with the International Standards for the Neurological Classification of Spinal Cord Injury (ISNCSCI) and the Spinal Cord Independence Measure (SCIM). The patients also completed the self-administered Neuropathic Pain Symptom Inventory (NPSI), and each patient’s short-latency somatosensory evoked potential (SSEP) was determined. The patients then underwent an assessment for the detection of the dysesthesia-matched frequency and intensity of electrical stimulation, i.e., the dysesthesia-matched TENS (DM-TENS) (Details of this assessment are described below in see section “Assessment for dysesthesia-matched transcutaneous electrical nerve stimulation”). After 2–3 days had passed to reduce the influence of this evaluation, the patients were assessed as the control condition by the Short-Form McGill Pain Questionnaire version-2 (SF-MPQ2) and by quantitative sensory testing (QST) without TENS. In the subsequent TENS interventions, DM-TENS and conventional high-frequency TENS (HF-TENS) were randomly administered on separate days for the TENS washout period.

**FIGURE 1:**
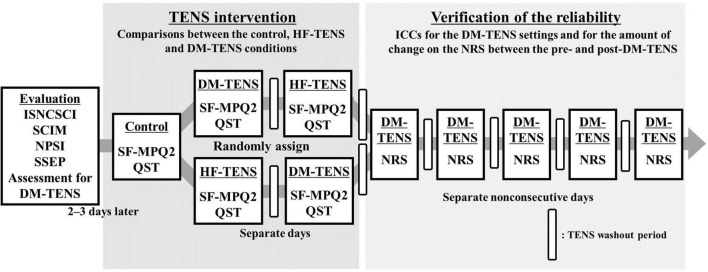
Flow diagram of the experimental procedures. Assessments of the patients’ spinal cord injury symptoms and an assessment for the detection of the DM-TENS setting were conducted. Next, in the TENS interventions, DM-TENS and conventional HF-TENS were administered on separate days, randomly. The patients were each subjected to the DM-TENS settings five times on separate non-consecutive days to determine the reliability of the DM-TENS settings. ISNCSCI, the International Standards for the Neurological Classification of Spinal Cord Injury; NPSI, the self-administered Neuropathic Pain Symptom Inventory; NRS, the numeric rating scale ranging for the degree of the subjective dysesthesia; QST, quantitative sensory testing; SCIM, Spinal Cord Independence Measure; SF-MPQ2, Short-Form McGill Pain Questionnaire version-2; SSEP, short-latency somatosensory evoked potential.

After the TENS interventions, for the verification of the reliability of the DM-TENS settings and the effects of the DM-TENS on dysesthesia, the patients were each subjected to the DM-TENS settings five times on separate non-consecutive days for the TENS washout period (Figure 1).

### Assessments for the symptoms of spinal cord dysfunction

The ISNCSCI provides classifications of patients’ neurologic levels, motor levels, and sensory levels and the degree of severity of the spinal cord injury (SCI) according to the ASIA Impairment Scale as a neurologic evaluation (Betz et al., 2019). The motor scores evaluate the strength of each muscle bilaterally, and the sensory scores evaluate the subject’s sensitivity to pin prick (PP) and light touch (LT) in each dermatome. The SCIM is a comprehensive assessment of daily function in patients with an SCI (Catz et al., 2007; Itzkovich et al., 2007).

The self-administered NPSI evaluates the following pain symptoms: superficial and deep spontaneous ongoing pain, brief pain attacks or paroxysmal pain, evoked pain, and abnormal sensations. The temporal items assessing the duration of spontaneous ongoing pain and the number of pain attacks were not analyzed in this study.

Short-latency somatosensory evoked potential are brain and spinal cord responses elicited by sensory stimuli. Here, the patients’ SSEPs were measured following a standardized protocol (American Clinical Neurophysiology Society, 2006). A transcutaneous electrical nerve stimulus was delivered to the median or ulnar nerves at the wrist according to the area where the patient’s dysesthesias were most severe. We calculated the latency and the amplitude of the N20 responses. The N20 responses (the cortical response) are related to impairments of the somatosensory pathway (Ozdemir and Perez, 2018).

The SF-MPQ2 consists of 22 sensory items and four affective items; it assesses a patient’s pain characteristics (Dworkin et al., 2009). We were interested specifically in the sensory qualities associated with pain.

The QST assesses the somatosensory function of skin and deep somatosensory afferents. We used the bedside-QST with the easy-to-use bedside device reported by Reimer and colleagues to assess the immediate effects of TENS (Baron et al., 2017; Reimer et al., 2020). The QST measured the following items in reference to the methods reported by Reimer et al. (2020) cold and heat detection, cold and heat pain detection, dynamic and static mechanical detection, mechanical pain sensitivity, wind-up, dynamic mechanical allodynia, pressure pain sensitivity, and vibration detection. The patients’ perception intensity was measured at the test area in a comparison with the infraorbital facial area on a 21-point NRS from 0 to 20, where 10 is defined as the intensity of the cheek area (< 10: less intensity at the affected area compared to the cheek; >10: stronger intensity at the affected area compared to the cheek). Pain intensity was measured on an 11-point NRS ranging from 0 (“I feel something, but it is not painful, merely a sensation”) up to 10 (“This is the worst tolerable pain I can imagine”).

### Assessment for dysesthesia-matched transcutaneous electrical nerve stimulation

To conduct electrical stimulation (Espurge, Ito Physiotherapy and Rehabilitation Co., Tokyo) to the area of the highest intensity of dysesthesia in a patient’s hand, we attached 5-cm^2^ self-adhesive electrodes (Axelgaard Manufacturing, Fallbrook, CA, United States) at the wrist located over the medial or ulnar nerves. The stimulation settings were as follows: a continuous pulse pattern, 50-μsec pulse duration, and a biphasic current with a symmetrical waveform.

For the detection of the dysesthesia-matched frequency and intensity of electrical stimulation, each patient’s perception was tested with electrical stimulation from 10 to 120 Hz at various frequencies and from the patient’s sensory threshold to 19 mA above the threshold at various intensities. The electrical perceptual threshold of each patient was measured by using single-frequency transcutaneous electrical nerve stimulation. In a case in which the sensory threshold was higher than the motor threshold, the electrical perceptual threshold from 19 mA below the motor threshold to the patient’s motor threshold was tested. Specifically, each patient received electrocutaneous stimuli that increased in 1-mA steps at each frequency. The patient then described the relative intensity of spontaneous dysesthesia compared to the dysesthesias evoked by electrical stimuli on a seven-point Likert scale as follows: −3: “much stronger feeling of dysesthesia than the electrical stimulation,” −2: “feeling the dysesthesia more than the electrical stimulation,” −1: “slight feeling of dysesthesia compared to the electrical stimulation,” 0: “the same intensity of dysesthesia and the electrical stimulation but they are “distinguishable,”” 1: “slightly greater feeling of the electrical stimulation than the dysesthesia,” 2: “feeling the electrical stimulation more than the dysesthesia,” and 3: “much stronger feeling of the electrical stimulation than the dysesthesia.” Spontaneous dysesthesias such as tingling and numbness are perceived intermittently at short temporal intervals, like the heartbeat. The patient was then asked to describe whether the frequency of the electrical stimulation was low, high, or matched compared to the beats of the spontaneous dysesthesia. To prevent bias in associating this assessment with DM-TENS, the patients were not told that this assessment was for DM-TENS. The parameters of the dysesthesia-matched TENS (DM-TENS) were set at (i) a stimulus intensity of 0 on the seven-point Likert scale, (ii) a frequency that matched the beats of the spontaneous dysesthesias, and (iii) the lowest spontaneous dysesthesias. There were no cases in which muscle contraction occurred during electrical stimulation.

### Transcutaneous electrical nerve stimulation interventions

Before the TENS interventions, the control condition was evaluated at the patients’ baseline (i.e., Control condition) without TENS and was assessed by the SF-MPQ2 and QST in the areas where the dysesthesias were the most severe. The DM-TENS and the conventional HF-TENS were administered on separate days, and their order was randomly assigned to each patient by a computer program; the patients were blinded to these assignments. The patients received each type of TENS in a 60-min session and were then assessed with the SF-MPQ2 and QST.

The HF-TENS was set as a continuous pulse pattern with a 400-μsec pulse duration, a biphasic current with a symmetrical waveform, at 100-Hz frequency, and at twice the intensity of the patient’s sensory threshold (Gewandter et al., 2019).

### The reliability of the dysesthesia-matched transcutaneous electrical nerve stimulation settings and the effects of the dysesthesia-matched transcutaneous electrical nerve stimulation on dysesthesia

After the TENS interventions, based on the intensity and frequency of the DM-TENS, the patients were each subjected to the DM-TENS settings five times on separate non-consecutive days for the TENS washout period to determine the reliability of the DM-TENS settings. The intensity and frequency of the DM-TENS settings were recorded. The patients answered the subjective questions about dysesthesia on the 11-point NRS before and after DM-TENS. The amount of change in subjective dysesthesia on the NRS between the pre- and post-DM-TENS was calculated each time for each patient.

### Statistical analyses

We used the Wilcoxon signed-rank test for pairwise comparisons to compare the patients’ SF-MPQ scores on the 22 items and the QST results between the control, HF-TENS, and DM-TENS conditions in a single session of the TENS intervention (i.e., the DM-TENS intervention was analyzed for only the first single session). These analyses were corrected for multiple comparisons by Holm corrections (Holm, 1979). To evaluate the reliability of the DM-TENS settings and the effects on dysesthesia, we determined the intraclass correlation coefficients (ICCs) for the intensity and frequency of the DM-TENS settings and the amount of change in subjective dysesthesia on the NRS between the pre- and post-DM-TENS on five separate non-consecutive days, not including the session of DM-TENS for TENS intervention. The R ver. 4.1.0 software program was used for statistical processing, and the level of significance was set at *p* < 0.05.

## Results

**FIGURE 2:**
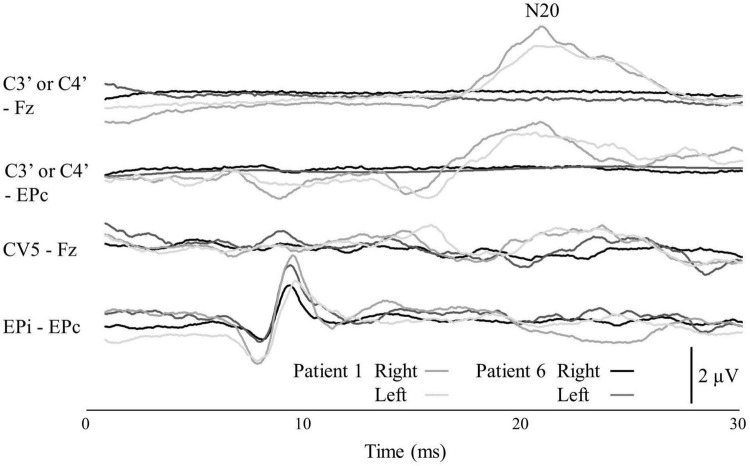
The SSEP of Patients 1 and 6. The locations of the four recording electrode pairs were: (1) the scalp overlying the contralateral somatosensory cortex (C3′ or C4′) according to the international 10–5 system and front parietal zone (Fpz); (2) C3′ or C4′ and the contralateral Erb’s point (EPc); (3) the C5 spinous process and the Fpz; and (4) the ipsilateral Erb’s point (EPi) and EPc. The top channel shows the N20 potential.

Table 1 summarizes the demographic and clinical data of the nine patients with dysesthesia due to spinal cord dysfunction. The SSEP responses of N20 in Patient 6 were not identified (Figure 2).

**TABLE 1:** Clinical features of the nine patients.

No.	Sex	Age	Diagnosis	Duration (months)	ASIA	Level of injury	UEMS	LEMS	LT	PP	SCIM	Side	NPSI	SSEP N20	
														Latency (ms)	Amplitude (μV)
P1	M	78	SCI at C2-C5	14	D	C3	42	38	62	66	85	Right	12	21.1	2.09
												Left	10	21.3	1.46
P2	F	72	SCI at C4-C6	6	D	C5	45	38	64	80	82	Right	31	20.5	0.80
												Left	14	20.5	1.05
P3	F	74	AAS	10	D	C1	38	30	58	72	65	Right	4	21.5	1.16
												Left	8	20.5	0.65
P4	M	84	SCI at C3-C7	4	D	C5	48	44	94	96	90	Right	22	Missing	
												Left	22	Missing	
P5	F	74	CSM at C3-C5	4	D	C5	39	42	62	109	79	Right	16	22.0	1.19
												Left	12	20.5	1.16
P6	M	69	SCI at C3-C5	6	C	C3	16	12	38	38	12	Right	35	Absent	
												Left	35	Absent	
P7	M	76	CSM at C4-C5	6	D	C4	48	38	101	108	74	Right	4	19.9	0.58
												Left	0	19.2	1.34
P8	F	55	TM at C6-C7	40	D	C6	48	35	66	72	90	Right	0	20.2	1.74
												Left	69	21.4	0.46
P9	F	88	CSM at C3-C6	5	D	C3	36	35	93	106	54	Right	16	20.9	0.75
												Left	14	21.0	0.85

The SSEP data of Patient 4 are missing, and the N20 of Patient 6 is absent. AAS, atlanto-axial subluxation; CSM, cervical spondylotic myelopathy; F, female; LEMS, lower-extremity motor score; LT, sensitivity to light touch; M, male; PP, sensitivity to pin prick; SCI, spinal cord injury; SCIM, Spinal Cord Independence Measure; TM, transverse myelitis; UEMS, upper-extremity motor score.

### Dysesthesia-matched transcutaneous electrical nerve stimulation settings

**FIGURE 3:**
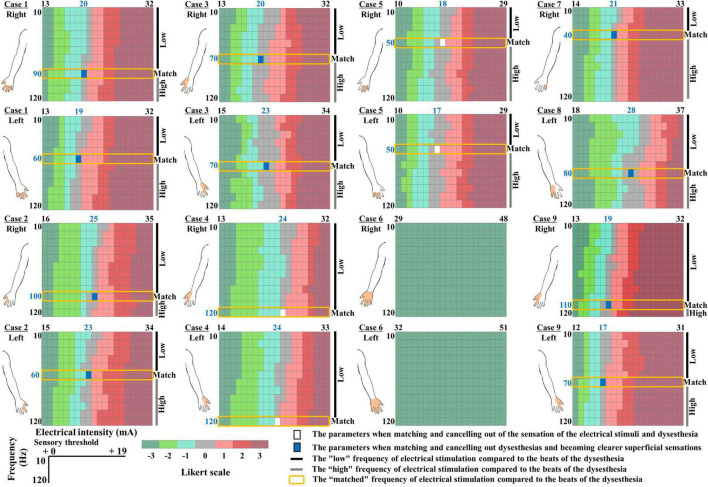
Color map depicting the relationship between dysesthesias and each intensity of electrical stimulation. Seven-point Likert scale as follows: -3: “much stronger feeling of dysesthesia than the electrical stimulation,” -2: “feeling the dysesthesia more than the electrical stimulation,” -1: “slight feeling of dysesthesia compared to the electrical stimulation,” 0: “the same intensity of dysesthesia and the electrical stimulation but they are “distinguishable,”” 1: “slightly greater feeling of the electrical stimulation than the dysesthesia,” 2: “feeling the electrical stimulation more than the dysesthesia,” and 3: “much stronger feeling of the electrical stimulation than the dysesthesia.” *Color bar:* A seven-point Likert scale of the relationship between dysesthesia and each intensity of electrical stimulation. *Filled white area:* The parameters when the intensity and frequency of the electrical stimulation match the dysesthesia profile. *Filled blue area*: The parameters that canceled out dysesthesias, and superficial sensations became clearer. *Blue fonts*: The dysesthesia-matched intensity and frequency of the electrical stimulation. The *filled orange area* in the illustration of the upper limb indicates the area of dysesthesia.

The dysesthesia occurred in both hands of six patients, the right hand of one patient, and the left hand of two patients. We were able to set the electrical stimulation that was consistent with the patient’s subjective dysesthesia for eight of the nine patients. The remaining subject, Patient 6, was unable to undergo TENS due to severe sensory deficits in which the motor thresholds preceded the sensory thresholds (Figure 3). Fourteen hands of eight patients were thus analyzed. All of the patients except Patient 6 reported that spontaneous dysesthesia by spinal cord dysfunction and dysesthesia by electrical stimuli coexisted. None of the patients reported that the electric stimulation made the dysesthesia more intense, and Patients 2, 5, and 8 reported that the electric stimulus was replaced by spontaneous dysesthesia when the seven-point Likert scale score was 3.

In all of the patients except Patient 6, there was a Likert scale of 0, i.e., the stimulus intensity of the electrical stimulation matched the intensity of the dysesthesia, at all frequencies of the electrical stimulation. The match between the frequency of the electrical stimulation and the beats of the spontaneous dysesthesia was the consistent value regardless of the stimulation intensity. Interestingly, After the patients clearly reported the intensity and frequency of the electrical stimulation matched the spontaneous dysesthesia, the sensations of the electrical stimulation and dysesthesia diminished and canceled each other (Figure 3, filled white area). The blue font in Figure 3 indicates the dysesthesia-matched intensity and frequency of the electrical stimulation.

Patients 1, 2, 3, 8, and 9 said that during the HF-TENS and during the control condition (baseline), their hand’s sensations were poor (as if they were wearing gloves), but with DM-TENS, the numbness sensation was canceled out and the superficial sensation became clearer (Figure 3, filled blue area). The patients also said that during the HF-TENS, the electrical stimulation modified their hands’ sensations compared to the sensation experienced during the DM-TENS. In four of the six patients with dysesthesia in both hands, the frequency of the electrical stimulation was identical in both hands, but it differed between the left and right hands in Patients 1 and 9 (Figure 3).

### Short-Form McGill Pain Questionnaire version-2, quantitative sensory testing, and tingling or numbness

**FIGURE 4:**
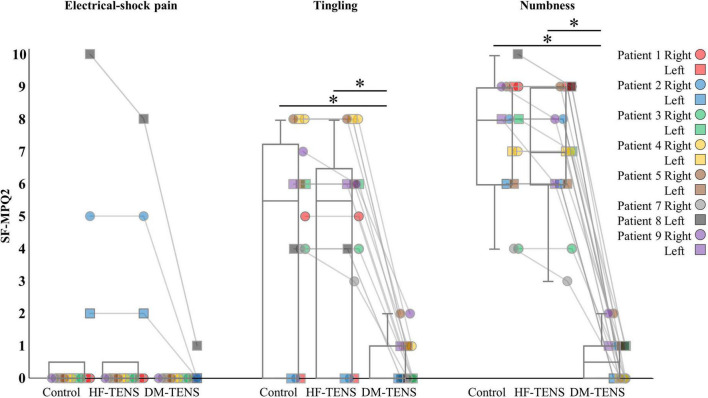
The specific items of the SF-MPQ-2 for each patient. HF-TENS: high-frequency TENS, DM-TENS: dysesthesia-matched TENS. *p < 0.05.

**FIGURE 5:**
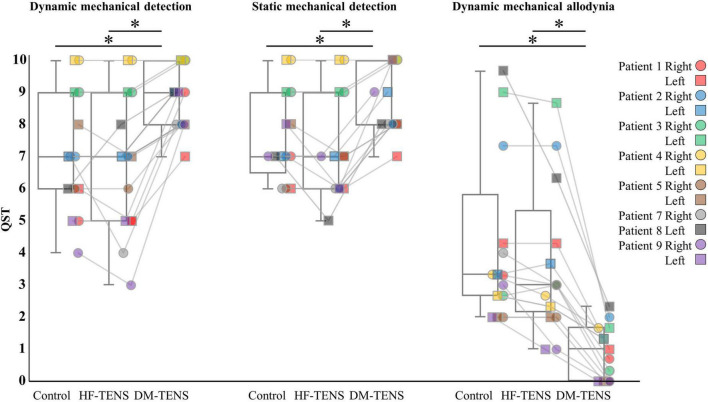
The specific items of the QST for each patient. *p < 0.05.

**FIGURE 6:**
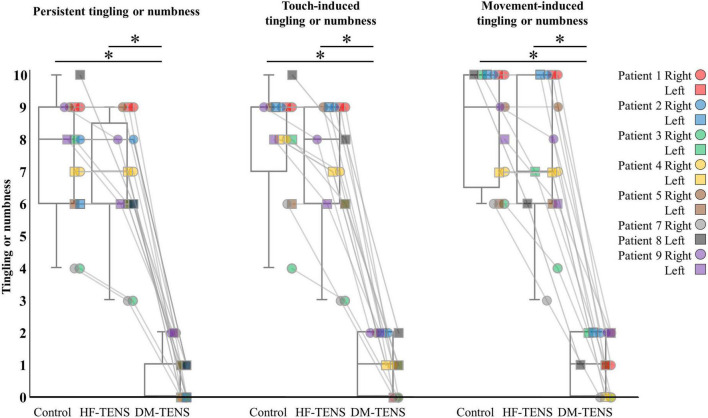
The specific items of the tingling or numbness symptom for each patient. *p < 0.05.

Figures 4–6 demonstrate the patients’ responses to the specific items of the SF-MPQ2 (item 16, electrical-shock pain; item 21, tingling; item 22, numbness), QST (item 9, dynamic mechanical detection; item 10, static mechanical detection; item 13, dynamic mechanical allodynia) and a questionnaire about symptoms of tingling or numbness. Supplementary Tables 1, 2 present all items of these questionnaires. In the SF-MPQ2, the sensations of tingling or pins and needles were significantly decreased in the DM-TENS condition, and the numbness sensation was significantly decreased compared to the control (*p* < 0.05) and HF-TENS (*p* < 0.05) conditions (Figure 4 and Supplementary Table 1).

The patients’ responses to the QST revealed that the DM-TENS significantly improved the dynamic and static mechanical hypoesthesia and the dynamic mechanical allodynia compared to the control (*p* < 0.05) and HF-TENS (*p* < 0.05) conditions (Figure 5 and Supplementary Table 2). The application of DM-TENS also significantly improved the persistent, the touch-induced, and the movement-induced tingling or numbness sensations compared to the control (*p* < 0.05) and HF-TENS (*p* < 0.05) conditions (Figure 6).

### Reliability of the dysesthesia-matched transcutaneous electrical nerve stimulation settings

Regarding the reliability of the DM-TENS settings, our analyses revealed that the ICC (1,5) of the intensity of DM-TENS was 0.95 (*p* < 0.01); the ICC (1,5) of the frequency of DM-TENS was 1.00 (*p* < 0.01), and the ICC (1,5) of the effect of the DM-TENS on dysesthesia was 0.98 (*p* < 0.01) (Supplementary Table 1).

## Discussion

In this study, we propose the use of dysesthesia-matched transcutaneous electrical nerve stimulation (DM-TENS) as a novel TENS treatment for dysesthesia in patients with spinal cord dysfunction. We conducted this study to verify the immediate effects of DM-TENS from the neuropathological perspective in an in-depth case series. Most of the study’s nine patients had bilateral disorders, but Patients 7 and 8 had unilateral disorders. Most of patients had only dysesthesia, but Patients 2 and 8 had both dysesthesia and pain (Figure 4 and Supplementary Table 1).

All patients except Patient 6 (with severe somatosensory deficit) reported the intensity and frequency of the electrical stimulation matched the spontaneous dysesthesia; thus the electrical stimulation was induced the sensations of the dysesthesia. After that, interestingly, the sensations of the electrical stimulation and dysesthesia diminished and canceled each other. Therefore, the effect of canceling out occurred as a result that the electrical stimulation induced the sensations of the dysesthesia. In Patient 6, considering the absent N20 of the SSEP, the DM-TENS was not adaptable because the somatosensory pathways between the spinal cord and the somatosensory cortex were severely impaired by the patient’s spinal cord injury, and the perception of TENS was thus difficult. A patient’s N20 response of the SSEP may therefore be a clue to determine his or her adaptation to DM-TENS.

Although the intensity and frequency of the DM-TENS were not consistent among the present patients, both the intensity and frequency of the DM-TENS and the degree of improvement of the dysesthesia by DM-TENS were reliable within the individual patients.

In Patients 1 and 9, the frequencies and the intensities of electrical stimulation were different between the right and left sides. In the other patients, the frequencies were identical, but the intensities differed between the two sides. The evaluation and expression of the dysesthesias also differed between the sides, indicating that it is necessary to evaluate the dysesthesias and set the DM-TENS for each side of a patient’s body.

Interestingly, we observed that the electrical stimulations gave the patients a mixed sensation of electrical stimulation and dysesthesia, but when the intensity and frequency of the electrical stimulation matched the patient’s subjective dysesthesia, the electrical stimulation and dysesthesia diminished each other or canceled each other out. This phenomenon occurred even though the intensity of the electrical stimulation was higher than the patients’ sensory thresholds. The persistent, touch-induced, and movement-induced tingling or numbness were significantly improved by DM-TENS compared to the control and conventional (high frequency [HF])-TENS conditions. The effects of the DM-TENS on numbness were also clearly greater than those of the conventional TENS for the peripheral neuropathic pain reported in an earlier study (Gewandter et al., 2019).

The general mechanisms of TENS as a neuromodulatory approach are thought to involve both peripheral nervous system and CNS mechanisms (Vance et al., 2014). In the peripheral mechanism, the antidromic activation of peripheral nerves (i.e., Aβ fibers) by TENS generates a peripheral blockade of nociceptive impulses, since antidromic nerve impulses would collide with and inhibit afferent impulses arising from peripheral structures (Johnson, 2007). On the other hand, in the CNS mechanism, activation of the inhibitory interneurons in the dorsal horn of the spinal cord by TENS reduces the firing rate of the projection neurons, and thus nociceptive impulses are prevented from traveling to the CNS in accord with the “gate control theory” (Melzack and Wall, 1994; Kasat et al., 2014). In addition, TENS induces the recruitment of the descending pain inhibition system that is related to the neuronal activity of the periaqueductal gray, rostral ventromedial medulla, and spinal cord (Sluka et al., 1999; Kalra et al., 2001; DeSantana et al., 2008; Lin et al., 2020).

These TENS effects based on peripheral and central mechanisms with the TENS set to high intensity were reported for neuropathic pain, but conventional TENS was reported to induce paresthesia and mechanical hypoesthesia (Meyer-Frießem et al., 2019). However, our present findings demonstrated that DM-TENS not only improved the patients’ dysesthesias and mechanical hypoesthesia but also canceled out the sensation of electrical stimulation, which cannot be explained by conventional theories of the peripheral and central mechanisms underlying the neuromodulatory approach of electrical stimulation.

One of the possible mechanisms underlying the effect of DM-TENS on dysesthesias is a selective neural blockade of nerves associated with dysesthesia (i.e., the “busy line effect”). As anecdotal evidence, primarily the amplitude with some contribution from the pulse width determines the number of neural fibers recruited and results in a perceived increase or decrease in the intensity and/or area of paresthesia sensation. The frequency of somatosensory stimulation influences how often a neuron fires in response to a stimulus (Deer et al., 2019). Therefore, matching the intensity and frequency of electrical stimulation to the subjective intensity and beats of the spontaneous dysesthesia (i.e., DM-TENS) may be effective because the electrical stimulation matches the number of fibers and depolarizations of the dysesthesia and blocks the dysesthesia-specific nerve fibers, but not other nerve fibers (including somatosensory nerve fibers). The DM-TENS (and HF-TENS) settings that we used in this study could not directly block Aδ fibers, as this would require much higher intensity than that typically provided by TENS (Levin and Hui-Chan, 1993). A busy line effect by DM-TENS may thus occur only in Aβ fibers.

However, the present study did not produce neurophysiological data supporting this possibility. Further investigations are necessary to clarify the neural mechanisms underlying the improvement of dysesthesias by DM-TENS. Based on previous observations that numbness contributes to sensory loss, the improvement in numbness by DM-TENS may have resulted in sensory acuity, and the residual sensory loss may represent a sensory loss of intrinsic disease origin.

We also observed that dynamic mechanical allodynia was significantly improved by DM-TENS compared to the control and HF-TENS conditions, although the cold and heat hyperalgesia showed no significant differences among the three conditions. Most of our patients did not have cold or heat hyperalgesia, but the hyperalgesia of Patients 2, 5, and 8 was improved by DM-TENS. Additional studies are thus needed to clarify the effect of DM-TENS on cold and heat hyperalgesia. Conventional TENS was reported to be effective against allodynia and thermal hyperalgesia (Tong et al., 2007; DeSantana et al., 2008). DM-TENS may affect these two types of pain to different degrees (Torebjork et al., 1984; Magerl et al., 2001). DM-TENS had no effect on Patient 6 (for whom the N20 was not derivable in the SSEP), suggesting that the dysesthesia-reduction effect may be involved in cortical processing and is unlikely to be a blocking effect at the spinal level (i.e., gate control effects). DM-TENS may modify the signal-to-noise ratio in cortical processing. Future studies should clarify these mechanisms by verifying carryover effects of DM-TENS and by intervening in the cases of patients with central or peripheral nerve disease and dysesthesias.

Several study limitations should be considered when interpreting the present findings. Our results should be interpreted with caution, and their generalizability remains unclear due to the design of this study. We contend that this case series study is better in terms of the evaluation and validity of treatment effects on dysesthesia, because between-group comparisons cannot reveal the highly individualized profiles of the dysesthesias; the characteristics of our novel TENS (DM-TENS) focus on that individualization. In addition, the effects of different pulse duration have been unknown under the comparative conditions of this study. We did not verify the pulse duration in the dysesthesia-matching process, because the evaluation of three factors of TENS (i.e., frequency, stimulus intensity, and pulse duration) requires a long time, and there is concern about severe patient fatigue, which may affect the accuracy of the evaluation. However, adjustment of the pulse duration may affect the matching to the dysesthesia’s intensity by an induced depolarization of sensory fibers. The effects of different pulse duration on the sensory matching process may need to be investigated in the future.

Regarding the upper limit of the stimulus intensity in assessments for DM-TENS, a prolonged evaluation with intensities up to the upper limit could result in fatigue and muscle contraction-induced exhaustion. We also considered the possibility that sensation-evoked muscle contraction could adversely affect the effectiveness of TENS, and we thus kept the stimulus intensity below the motor thresholds. However, because Patient 6 could not perceive the electrical stimulation below the motor threshold, it appears that DM-TENS could be performed by setting the stimulation intensity at a value higher than a patient’s motor threshold.

Moreover, the data reported in the questionnaire were values characterizing sensations at the time that the patient was completing the questionnaire. Each TENS intervention was just one session, but the type, intensity, and location of sensations may vary over time. In this study, spontaneous dysesthesias were at the same location in the hands of all of the patients, without spatial variation. The dysesthesias of only Patient 5 were sometimes increased at midnight, but in the other patients, the type and intensity of dysesthesias were stable. Future studies should consider time and spatial variations of dysesthesias. For the present TENS interventions, DM-TENS and HF-TENS were administered on separate days, and their order was randomly assigned to each patient by a computer program; the patients were blinded to the treatments. However, the experimental design of this study cannot adequately remove a placebo effect. An added sham-TENS condition or a randomized controlled trial could robustly verify the effectiveness of DM-TENS for dysesthesias.

## Conclusion

The application of DM-TENS improved dysesthesias and mechanical hypoesthesia in patients with spinal cord dysfunction. The effectiveness of DM-TENS on the symptoms of tingling or numbness in particular were clearly higher and were reliable within the patients. DM-TENS may provide a new development in the treatment of dysesthesias that has not been systematically established. Our study’s preliminary efficacy results suggest that further neurophysiological evaluations of DM-TENS for dysesthesias in a variety of diseases are warranted.

## Data availability statement

The original contributions presented in this study are included in the article/Supplementary material, further inquiries can be directed to the corresponding authors.

## Ethics statement

The studies involving human participants were reviewed and approved by the Nishiyamato Rehabilitation Hospital. The patients/participants provided their written informed consent to participate in this study. Written informed consent was obtained from the individual(s) for the publication of any potentially identifiable images or data included in this article.

## Author contributions

YN and KI designed the study. YN analyzed the data and wrote the manuscript. YM and YI collected the data. MO and SM supervised the study. All authors have read and agreed to the published version of the manuscript.
