# Supplementary material for: A novel form of transcutaneous electrical nerve stimulation for the reduction of dysesthesias caused by spinal nerve dysfunction: A case series
Source: Front Hum Neurosci. 2022 Aug 24;16:937319. doi: 10.3389/fnhum.2022.937319 (PMC9449584; doi:10.3389/fnhum.2022.937319)
Supplement: Supplementary file 1 [file Table_1.docx]

**Suppl. Table S1.** The raw data of the reliability of the DM-TENS settings and effects on dysesthesia**.** The stimulus intensity and frequency of the DM-TENS settings and amount of change in subjective dysesthesia on the NRS between the pre- and post-DM-TENS. T1–5: from one to five times on separate days.
