# Supplementary material for: A novel form of transcutaneous electrical nerve stimulation for the reduction of dysesthesias caused by spinal nerve dysfunction: A case series
Source: Front Hum Neurosci. 2022 Aug 24;16:937319. doi: 10.3389/fnhum.2022.937319 (PMC9449584; doi:10.3389/fnhum.2022.937319)

**Suppl. Table S2.** The Short-Form McGill Pain Questionnaire version-2 (SF-MPQ-2) for each patient *p<0.05, Control vs. DM-TENS; #p<0.05, HF-TENS vs. DM-TENS.


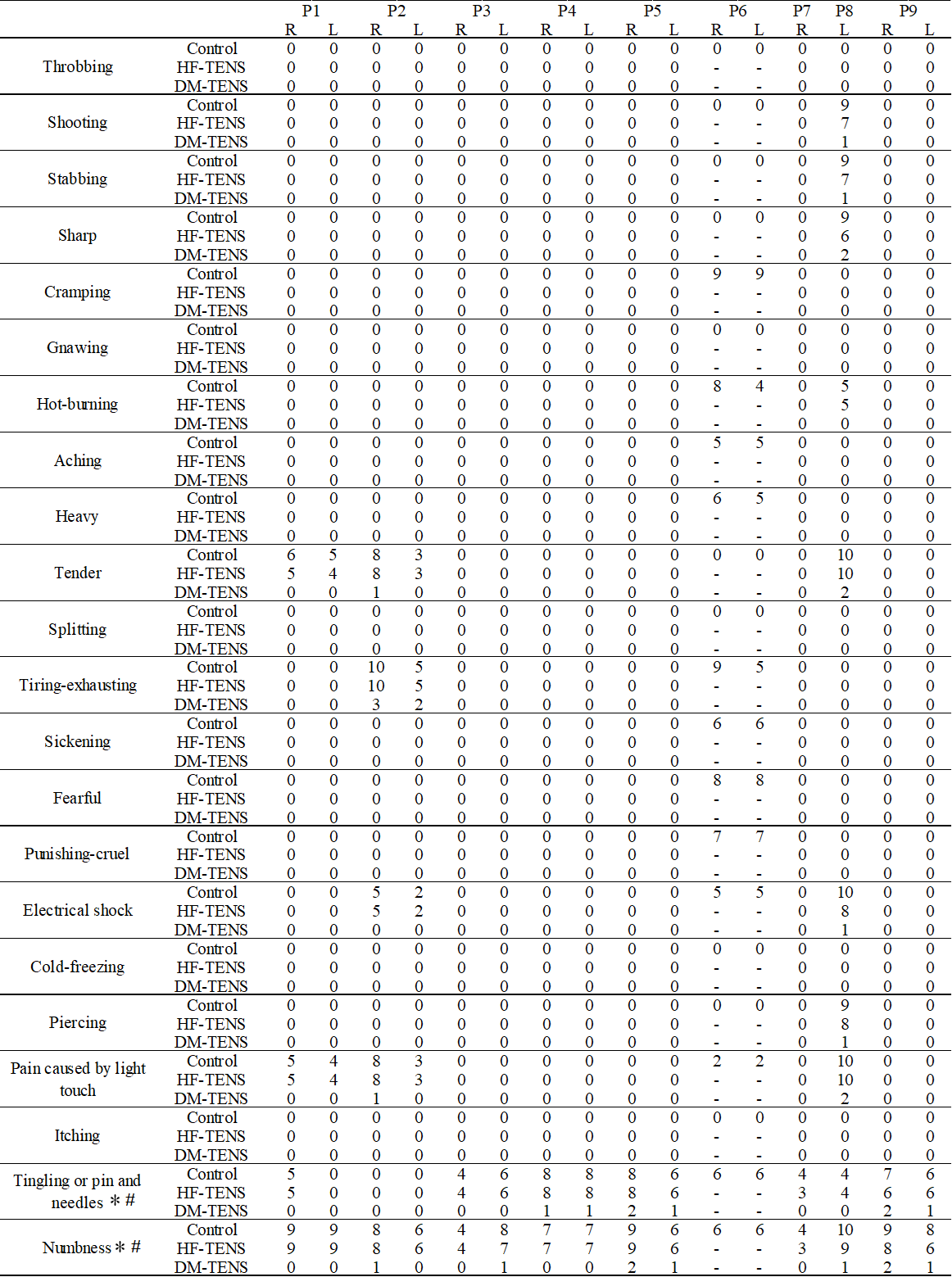

Supplement: Supplementary file 2 [file Table_2.docx]
